# Claims-Based Diagnostic Registration Patterns and Clinical Burden of Selected Common Aneuploidies in Korean Children: A Nationwide HIRA Study (2009–2023)

**DOI:** 10.3390/children13101360

**Published:** 2026-10-08

**Authors:** Hye Eun Kwon, So Hyeon Gwon, Nak-Hoon Son, Jun Chul Byun

**Affiliations:** 1 Department of Pediatrics, Gachon University Gil Medical Center, Incheon 21565, Republic of Korea; romani33@hanmail.net; 2 Department of Statistics, College of Natural Science, Keimyung University, Daegu 42601, Republic of Korea; gwonsohyeon@kmu.kr; 3 Department of Pediatrics, Keimyung University School of Medicine, Daegu 42601, Republic of Korea

**Keywords:** chromosome disorders, epidemiology, diagnostic registration, healthcare costs, child

## Abstract

**Highlights:**

**What are the main findings?**

- Nationwide HIRA data revealed distinct diagnostic registration patterns among five selected common aneuploidies in Korean children, with Down syndrome being the most frequently registered disorder.
- Edwards and Patau syndromes showed the greatest clinical and economic burden, with the highest in-hospital mortality, congenital heart disease prevalence, and first-year healthcare expenditures.

**What are the implications of the main findings?**

- Claims-based diagnostic registration measures should be distinguished from true cytogenetic birth prevalence, particularly for disorders with prenatal loss, early mortality, or delayed clinical recognition.
- Disorder-specific clinical pathways, integrated national surveillance, and targeted healthcare support may help address the substantial differences in medical and economic burden across chromosomal disorders.

**Abstract:**

Background: Selected common aneuploidies, including Down, Edwards, Patau, Turner, and Klinefelter syndromes, are important causes of congenital anomalies and diverse clinical morbidities. However, nationwide evidence regarding the recent diagnostic registration patterns, clinical outcomes, and healthcare burdens in South Korea remains limited. Methods: We conducted a nationwide descriptive analysis using claims data from the Health Insurance Review and Assessment Service (HIRA) of South Korea between 2009 and 2023. Among individuals aged 0–14 years, we evaluated period-based administrative registration frequency and ratios relative to live births, age at diagnosis, comorbidities including congenital heart disease (CHD) and epilepsy, in-hospital mortality, and first-year healthcare expenditures following the first observed claims-code date. Results: Among the 5,586,770 live births over the 15-year study period, 5900 individuals with one of the five selected common aneuploidies were identified in the HIRA database, corresponding to an overall claims-based diagnostic registration ratio of 105.6 per 100,000 live births. Down syndrome was the most frequently registered disorder, accounting for 59.51% of the cases, followed by Turner syndrome (19.56%), Klinefelter syndrome (14.76%), Edwards syndrome (4.63%), and Patau syndrome (1.54%). The corresponding claims-based diagnostic registration ratios were 62.8, 20.7, 15.6, 4.9, and 1.6 per 100,000 live births, respectively. Edwards and Patau syndromes had the highest clinical burden, with discharge-recorded in-hospital mortality of 34.07% (95% CI, 28.46–40.02) and 32.97% (95% CI, 23.47–43.61), respectively, and the highest median first-year healthcare expenditures of 17,394,435 KRW (Q1–Q3, 1,087,560–61,040,440) and 7,478,665 KRW (Q1–Q3, 510,510–37,524,080), respectively. The prevalence of CHD ranged from 60.7% to 70.3% in Down, Edwards, and Patau syndromes and was approximately 11% in Turner and Klinefelter syndromes. Conclusions: This nationwide claims-based study demonstrated distinct diagnostic registration patterns and outcome disparities among the selected common aneuploidies in Korean children. These findings support the need for disorder-specific clinical pathways, integrated national surveillance, and targeted supportive care to address the substantial clinical and economic burdens.

## 1. Introduction

Selected common aneuploidies, specifically the Down, Edwards, Patau, Turner, and Klinefelter syndromes, are the leading causes of congenital anomalies and pose a significant public health burden [1,2,3]. These five conditions represent the most frequent autosomal trisomies and sex chromosome aneuploidies. Despite their clinical importance, up-to-date nationwide data on claims-based diagnostic registration patterns, healthcare utilization, and outcome burden remain scarce. This lack of robust population-based longitudinal data hinders the formulation of evidence-based health policies and the efficient allocation of medical resources [1,2,3,4,5].

The natural history and healthcare needs of these chromosomal disorders differ substantially. Edwards and Patau syndromes are frequently associated with severe congenital anomalies and high mortality during infancy and early childhood [6,7], whereas Down syndrome is associated with substantial chronic morbidity, including a high burden of congenital heart disease, requiring continued medical follow-up [8,9]. Turner syndrome and Klinefelter syndrome generally have lower early mortality but may remain clinically unrecognized until childhood or adolescence because of less overt phenotypes and delayed clinical presentation [10,11,12,13,14,15]. These differing clinical trajectories may result in markedly different patterns of healthcare utilization and financial burden, ranging from intensive medical care early in life to prolonged multidisciplinary follow-up. Understanding these differences is important not only for clinical management but also for healthcare planning, resource allocation, and the development of appropriate support systems. Within the South Korean healthcare system, the government implemented the Rare Disease Copayment Reduction Program (RDCRP; San-jung-teuk-rye) in 2004, providing substantial financial support and reducing the medical cost burden for patients. Because inclusion in this program offers significant economic benefits, it heightens clinician awareness and necessitates precise diagnostic coding, thereby fostering a highly rigorous and systematic diagnostic environment. By leveraging this framework, we considered the initial identification of these five genetic disorders within the healthcare system as a point of clinical diagnostic registration. Although determining the exact biological onset of these disorders is clinically infeasible, this approach leverages a nationwide database to capture the earliest clinical presentation, providing a nationwide proxy for the timing of clinical recognition and diagnostic registration. Using these comprehensive data, we analyzed and compared claims-based diagnostic registration ratios and temporal patterns, comorbidities, mortality, and first-year healthcare expenditures for these five selected common aneuploidies among individuals aged 0–14 years from 2009 to 2023.

## 2. Methods

### 2.1. Data Source and Study Population

This retrospective observational study used customized national claims data from the Health Insurance Review and Assessment Service (HIRA) of South Korea from 2009 to 2023. To reduce left truncation, the study population was restricted to individuals born within or after 2009. Because exact dates of birth were unavailable in HIRA, birth year was estimated from the year of diagnosis and the recorded age at diagnosis. Individuals born before 2009 were excluded from the numerator because of left truncation: their births preceded the start of the observation period and are therefore not represented in the denominator. The tracking of these conditions aligns with their chronological inclusion in the RDCRP, which ensures standardized nationwide data collection. Down syndrome has been registered since 2004; Turner, Edwards, and Patau syndromes since 2009; and Klinefelter syndrome since 2019.

### 2.2. Disease Definitions and Clinical Outcomes

Selected common aneuploidies were identified using the following ICD-10 codes: Down syndrome (Q90.0–Q90.9); Edwards syndrome (Q91.0–Q91.3); Patau syndrome (Q91.4–Q91.7); Turner syndrome (Q96.0–Q96.9); and Klinefelter syndrome (Q98.0–Q98.4).

To examine administrative diagnostic registrations captured within the national healthcare system, a case was defined based on the first observed claims-code date—the earliest date on which an eligible disorder-specific ICD-10 code appeared in the HIRA database (incorporating both inpatient and outpatient settings, as well as principal and additional diagnosis fields, with no requirement for repeat coding) [16,17,18]. Because individual cytogenetic reports were unavailable for independent verification and no published validation study exists for this exact algorithm in HIRA data, we acknowledge that these codes may occasionally include provisional or incorrectly coded diagnoses, despite the physician verification required for the RDCRP. Consequently, this date represents the first administrative appearance of a code within our observation window rather than the precise date of clinical diagnosis or genetic confirmation. Because the HIRA claims database does not contain cytogenetic test results, prenatal diagnostic information, stillbirths, or pregnancy termination data, the estimates in this study were interpreted as claims-based diagnostic registration ratios rather than as true cytogenetic birth prevalence.

For each disorder, the overall claims-based diagnostic registration ratio was calculated by dividing the number of individuals with the corresponding ICD-10 code by the total number of live births during the study period and expressed per 100,000 live births. Annual trends were evaluated using the number of newly registered diagnoses in each calendar year, normalized to the number of live births in the same year, and were expressed as annual diagnostic registrations per 100,000 live births rather than birth-cohort incidence estimates. To evaluate chronological shifts, annual HIRA-based diagnostic registration trends were tracked during the study period. First-year healthcare expenditures were defined as the total medical costs incurred within 365 days of the first observed claims-code date. To ensure a complete 1-year follow-up period for cost estimation, patients whose first observed claims-code date occurred in 2023 were excluded from the first-year expenditure analyses.

Since Klinefelter syndrome was included in the RDCRP in 2019, diagnostic registration estimates prior to this period are likely subject to substantial under-ascertainment. Accordingly, the temporal trends in Klinefelter syndrome should be interpreted with caution. Given their significant clinical burden, we specifically analyzed the prevalence of epilepsy (G40 and G41) and congenital heart disease (Q20–Q26 and Q89.3) as key comorbidities within this cohort.

In-hospital mortality was defined as death recorded at discharge from the HIRA claims database during the study period. The disease-specific in-hospital mortality rates were calculated as the number of patients with discharge-recorded deaths divided by the number of patients with available discharge outcome records for each disorder. Therefore, these estimates represent discharge-recorded in-hospital mortality among patients with available discharge outcome information, and may not capture deaths occurring before diagnostic registration or outside hospital discharge records.

### 2.3. Statistical Analysis

Because the distribution of healthcare expenditures was strongly right-skewed, continuous variables, including first-year healthcare expenditures, are presented as the median with interquartile range (Q1–Q3) as well as the mean ± standard deviation (SD). Categorical variables, such as disease-specific in-hospital mortality and comorbidity frequencies, were expressed as frequencies and percentages with exact (Clopper–Pearson) 95% confidence intervals (CIs). This study was designed as a descriptive real-world evidence study; accordingly, no formal hypothesis testing was performed for between-disorder comparisons, and the reported contrasts should be interpreted as descriptive rather than as estimated effects. All statistical analyses were performed using SAS version 9.4 (SAS Institute Inc., Cary, NC, USA) and data visualizations were generated using R software version 4.3.0 (R Foundation for Statistical Computing, Vienna, Austria).

### 2.4. Ethics Statement

This retrospective observational study used de-identified administrative claims data obtained from the Korean Health Insurance Review and Assessment Service. The study protocol was reviewed and approved by the Institutional Review Board of Keimyung University Dongsan Medical Center (approval no. DSMC 2025-02-022-004). The requirement for informed consent was waived because only de-identified population-level data were analyzed. The study was conducted in accordance with the ethical principles of the Declaration of Helsinki.

## 3. Results

### 3.1. Epidemiological Characteristics

Over the 15-year study period, 5900 individuals with one of the five selected common aneuploidies were identified in the HIRA database, corresponding to an overall claims-based diagnostic registration ratio of 105.6 per 100,000 live births (yielding an administrative registration ratio of 1 per 947 live births) (Table 1). Down syndrome was the most frequently registered disorder, accounting for 59.51% of these cases (a registration ratio of 1 per 1591 live births), followed by Turner syndrome (19.56%; 1 per 4841 live births), Klinefelter syndrome (14.76%; 1 per 6414 live births), Edwards syndrome (4.63%; 1 per 20,464 live births), and Patau syndrome (1.54%; 1 per 61,393 live births).

Disorder-specific numerators and the annual number of live births used as the denominator are presented in Supplementary Table S1, and the corresponding birth-cohort–based estimates, obtained by assigning each case to its birth-year cohort, are presented in Supplementary Table S2.

Regarding sex distribution, the male-to-female ratio varied substantially across disorders. Among autosomal trisomies, Down syndrome showed a slight male predominance (54.8% males vs. 45.2% females). In contrast, both Edwards syndrome and Patau syndrome were more frequently identified in females (64.8% and 57.1%, respectively). For sex chromosome aneuploidies, the recorded sex distribution was highly concentrated, as expected; Turner syndrome cases were predominantly female (91.9%), while Klinefelter syndrome cases were almost exclusively male (99.5%). Ninety-three individuals with a Turner syndrome code were recorded as male (8.1% of 1154) and four individuals with a Klinefelter syndrome code were recorded as female (0.5% of 871); a sensitivity analysis excluding these sex-discordant records yielded essentially unchanged estimates for all principal outcomes (Supplementary Table S3).

Autosomal trisomies were typically identified at a younger age at diagnosis than sex chromosomal aneuploidies. The median age at diagnosis was 0.2 years for patients with Down, Edwards, and Patau syndromes, compared with 4.0 years for Turner syndrome and 0.9 years for Klinefelter syndrome. The means (SD) and Q1–Q3 values are presented in Table 1.

### 3.2. Epilepsy and Congenital Heart Disease

Regarding the prevalence of epilepsy, a clear divergence was observed across disorders. Patau syndrome and Edwards syndrome exhibited the highest rates, exceeding 10% at 16.5% (15/91) and 12.5% (34/273), respectively. In the remaining three disorders, the rates remained below 5%, but still represented a notable clinical presence. Down syndrome presented an epilepsy rate of 3.3% (116/3511), followed by Turner syndrome at 2.2% (25/1154), and Klinefelter syndrome at 1.6% (14/871).

The cardiovascular burden is even more pronounced, particularly among autosomal trisomies, where the prevalence of congenital heart disease (CHD) ranges from 60.7% to 70.3%. Patau syndrome had the highest prevalence (70.3%, 64/91), followed by Edwards syndrome (68.9%, 188/273) and Down syndrome (60.7%, 2130/3511). In contrast, patients with sex chromosome aneuploidies showed lower but consistent CHD rates, with Turner syndrome at 11.4% (132/1154) and Klinefelter syndrome at 11.3% (98/871).

### 3.3. Comparative Analysis of First-Year Healthcare Expenditures

Healthcare expenditure during the first year following the first observed claims-code date differed substantially across the five disorders (Table 1). Because the cost distribution was strongly right-skewed, the results are described primarily in terms of the median and interquartile range, with mean-based contrasts reported alongside them.

Compared with Klinefelter syndrome, which had the lowest expenditure (median 242,990 KRW; Q1–Q3, 85,015–453,780; mean 1,503,182 KRW), all other chromosomal disorders showed substantially higher healthcare utilization. The highest economic burden was associated with Edwards syndrome, with a median first-year expenditure of 17,394,435 KRW (Q1–Q3, 1,087,560–61,040,440), approximately 72 times that of Klinefelter syndrome. This was followed by Patau syndrome (median 7,478,665 KRW; Q1–Q3, 510,510–37,524,080; approximately 31 times) and Down syndrome (median 2,898,565 KRW; Q1–Q3, 521,720–13,467,270; approximately 12 times). Among sex chromosome aneuploidies, expenditure for Turner syndrome was modestly higher than that for Klinefelter syndrome (median 555,270 KRW; Q1–Q3, 222,890–2,957,070; approximately 2.3 times). When based on mean expenditures, the corresponding ratios relative to Klinefelter syndrome (mean 1,503,182 KRW) were 35.3 (52,998,814 KRW), 25.2 (37,897,745 KRW), 8.3 (12,455,946 KRW), and 1.9 (2,853,011 KRW), respectively.

### 3.4. Diagnostic Registration Trends of Selected Common Aneuploidies

**Figure 1:**
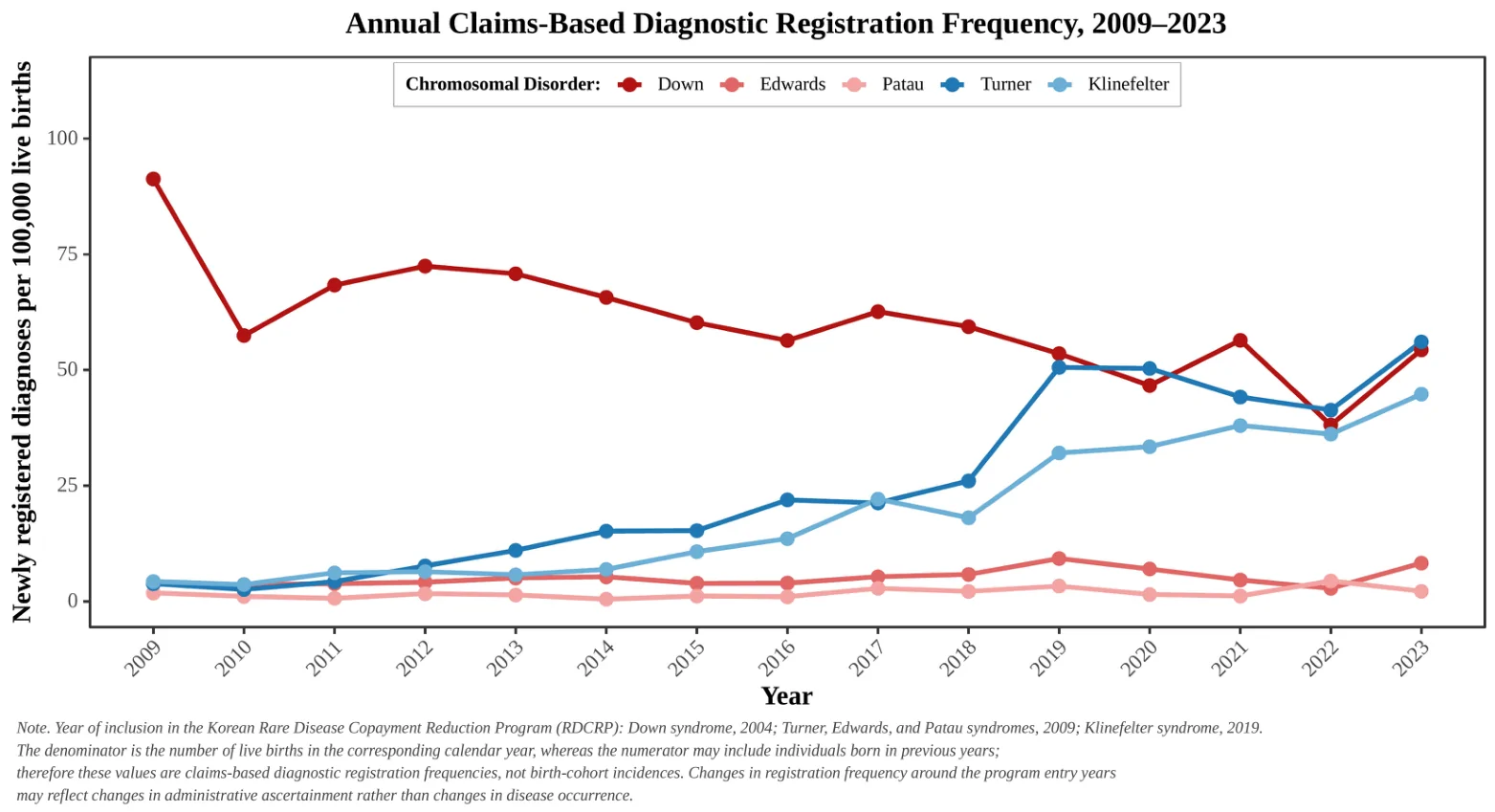
Annual Claims-Based Diagnostic Registration Frequency of Selected Common Aneuploidies in South Korea, 2009–2023. For each calendar year, the denominator is the number of live births in that year, whereas the numerator is the number of individuals with a first observed disorder-specific claims code in that year and may include individuals born in previous years. Therefore, these values represent annual claims-based diagnostic registrations normalized to contemporaneous live births rather than birth-cohort incidence estimates.

Figure 1 illustrates the annual diagnostic registration frequency (expressed per 100,000 live births) of the five selected common aneuploidies among individuals aged 0–14 years between 2009 and 2023. Down syndrome exhibited an overall downward trend despite transient fluctuations over the study period. In contrast, the diagnostic registration frequency for Turner syndrome and Klinefelter syndrome demonstrated a distinct upward trajectory, beginning in the mid-2010s. The annual diagnostic registration frequency for Edwards syndrome and Patau syndrome remained relatively stable throughout the study period and were characterized only by modest year-to-year variations.

### 3.5. Mortality of Five Chromosomal Disorders

**Figure 2:**
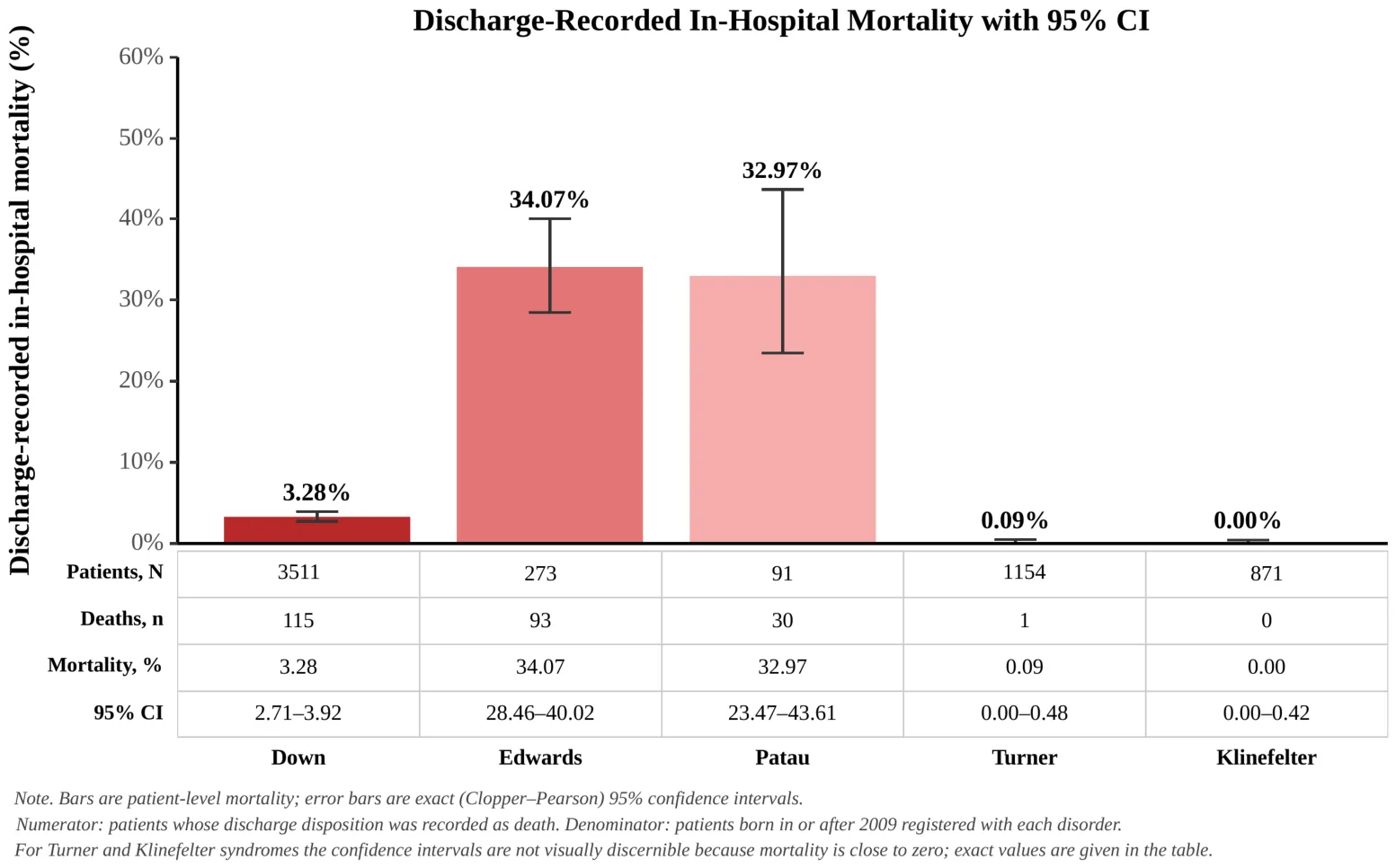
Discharge-Recorded In-Hospital Mortality Proportions Among Patients with Available Discharge Outcome Records, 2009–2023. The different colors represent the five aneuploidy groups shown in the figure.

Figure 2 shows patient-level discharge-recorded in-hospital mortality during the study period. Edwards syndrome and Patau syndrome had the highest mortality, at 34.07% (93/273; 95% CI, 28.46–40.02) and 32.97% (30/91; 95% CI, 23.47–43.61), respectively. Down syndrome showed a substantially lower mortality of 3.28% (115/3511; 95% CI, 2.71–3.92), whereas the sex chromosome aneuploidies showed minimal or no discharge-recorded in-hospital mortality, at 0.09% (1/1154; 95% CI, 0.00–0.48) for Turner syndrome and 0.00% (0/871; 95% CI, 0.00–0.42) for Klinefelter syndrome. Because deaths occurring outside a healthcare facility are not captured in claims data, these figures represent lower bounds on true mortality.

## 4. Discussion

Using the nationwide HIRA database, this study provides a comprehensive population-based estimate of the claims-based diagnostic registration burden of five selected common aneuploidies in South Korea. To our knowledge, this is the first study to simultaneously evaluate selected common aneuploidies within a single pediatric birth population using a national claims database.

Among the 5,586,770 live births recorded during the 15-year study period, these five disorders were identified in 5900 individuals, corresponding to an overall claims-based diagnostic registration ratio of 105.6 per 100,000 live births (1 per 947 live births). Notably, the observed claims-based diagnostic registration burden for all five conditions was consistently lower than the established global estimates of cytogenetic or live birth prevalence. The magnitude of this divergence between our findings and international estimates varied substantially across the five disorders, likely reflecting distinct clinical presentations, diagnostic timelines, and underlying biological mechanisms [1,2,3,4,5,10,11,19,20,21,22].

In the disorder group, the largest divergence between our findings and international data was observed for Klinefelter syndrome, for which the claims-based diagnostic registration ratio was 1 per 6414 live births. This rate is approximately six- to ten-fold lower than the global birth prevalence estimates of 1 in 500–1000 live births. This diagnostic gap likely reflects the subtle clinical phenotype of Klinefelter syndrome during the neonatal and pediatric periods, leading to a substantial under-ascertainment in claim-based pediatric databases. Furthermore, because our study population was limited to individuals younger than 15 years (born in or after 2009), pediatric claim-based ascertainment inevitably missed a substantial proportion of cases that are diagnosed later in adulthood, often during fertility evaluations [12,13,14,15]. Similarly, Turner syndrome showed a claims-based diagnostic registration ratio of 1 per 4841 live births, which was lower than the established birth prevalence estimates of approximately 1 in 2000 to 2500 live births [10,11]. However, the discrepancy was less pronounced than that observed for Klinefelter syndrome, likely because Turner syndrome has more overt pediatric manifestations, such as short stature, which may prompt earlier clinical evaluation and diagnostic registration.

For autosomal trisomies, the claims-based diagnostic registration ratios of trisomy 13 and trisomy 18 were also substantially lower than global estimates. In this study, the registration ratios for trisomy 13 and trisomy 18 were 1 per 61,393 and 1 per 20,464 live births, respectively, which were four- to six-fold lower than the global estimates. In contrast, the gap for Down syndrome was relatively modest, approximately two-fold, compared to global figures [1,19,20,21,22]. Overall, this divergence was more pronounced in highly lethal trisomies, Edwards syndrome, and Patau syndrome than in Down syndrome.

Regarding the underlying mechanisms, we hypothesize that these discrepancies in the diagnostic registration frequency may be consistent with South Korea’s advanced prenatal screening infrastructure. The widespread utilization of high-resolution ultrasonography and noninvasive prenatal testing (NIPT) could facilitate the early identification of major structural anomalies and chromosomal imbalances [23,24,25,26,27,28]. Although the potential impact of unrecorded pregnancy terminations or selective terminations on live-born registration cannot be discounted [4,20], the HIRA dataset contains no individual-level information on prenatal screening, prenatal diagnosis, or pregnancy termination, and thus these explanations cannot be directly evaluated. Three distinct processes should be distinguished: probabilistic prenatal screening, including first-trimester screening, maternal serum screening, and cell-free DNA testing; definitive prenatal diagnosis through invasive testing, such as chorionic villus sampling or amniocentesis; and postnatal clinical recognition and diagnosis followed by administrative coding and registration. The HIRA database contains no individual-level information on the first two processes and captures only the claims-based administrative registration component of the third. Moreover, the first observed claims-code date does not necessarily correspond to the precise date of clinical diagnosis or genetic confirmation. Therefore, the present data do not permit direct inference regarding prenatal screening uptake, prenatal diagnostic confirmation, or their causal relationship with subsequent registration patterns. Furthermore, distinct clinical trajectories further differentiate these autosomal disorders. For highly lethal conditions, such as Edwards and Patau syndromes, a fraction of affected neonates likely succumb shortly after birth before receiving a formal clinical diagnosis, leaving them uncaptured in the claims data.

Consequently, further investigation is warranted to clarify the drivers of these discrepancies. Future studies incorporating prenatal screening results, pregnancy outcomes, stillbirths, and cytogenetically confirmed diagnoses are needed to distinguish the true birth prevalence from claims-based diagnostic registration ratios in administrative claims data [1,6,7,16,17,18].

Overall, our results provide a real-world snapshot of chromosomal abnormalities in South Korea. These findings underscore that claims-based diagnostic registration patterns are shaped by the complex interplay between diagnostic timing, prenatal intervention, and disorder-specific clinical visibility.

As evidenced by the median age at diagnosis, Down syndrome and Edwards syndrome are typically identified within the first year of life because of overt anomalies. In contrast, Turner syndrome and Klinefelter syndrome were diagnosed later, with median ages of 4.0 and 0.9 years, respectively. The low median age for Klinefelter syndrome should be interpreted in light of the study design: because follow-up was limited to individuals born in or after 2009 (maximum age, 14 years), diagnoses made in late adolescence or adulthood, when most cases are identified [12,13,14,15], could not be captured. This disparity potentially stems from the differences in clinical visibility. For instance, Turner syndrome tends to be more frequently recorded during early to mid-childhood because of noticeable features, such as short stature, and the administrative requirement for a formal diagnosis to access insurance-covered growth hormone therapy [10,11]. In contrast, Klinefelter syndrome is often underreported during the pediatric period, as its subtle symptoms frequently remain unnoticed until late adolescence or adulthood [12,13,14,15].

Our multidimensional analysis, encompassing epilepsy, CHD, first-year healthcare expenditures, and mortality, revealed substantial differences in observed claims-based clinical and healthcare utilization burden among the studied chromosomal disorders. These four claims-derived indicators are influenced by age, timing of diagnosis, survival, healthcare-seeking behavior, and coding practice, and do not constitute a validated composite measure of overall clinical severity. Despite the high rates of spontaneous intrauterine fetal demise, Edwards and Patau syndromes consistently exhibited the highest burden across all four indicators for live-born infants [6,7]. Down syndrome showed the next highest burden, while sex chromosome aneuploidies demonstrated the lowest. Notably, the CHD burden in Down and Turner syndromes was concordant with previous Korean, meta-analytic, and guideline-based reports [8,9,29]. Because the cost distribution was strongly right-skewed, first-year expenditures are described using the median and interquartile range, and comparisons based solely on mean costs should be interpreted with caution.

Collectively, these distinct medical burden profiles reflect the inherent postnatal phenotypic severity of each disorder. Furthermore, these outcomes are closely intertwined with advanced prenatal screening techniques, such as expanded cfDNA/NIPT, and disorder-specific clinical trajectories [23,24,25,26,27,28], which collectively shape claims-based diagnostic registration patterns and subsequent healthcare utilization in South Korea.

Longitudinal trends from 2009 to 2023 (Figure 1) reflect the shifting landscape of prenatal diagnostics, administrative policies, and clinical recognition in South Korea. It is plausible that the overall decline in Down syndrome, despite the rising maternal age, may be related to the expanded use of prenatal screening and subsequent alterations in diagnostic registration. However, the HIRA data cannot directly evaluate these mechanisms or distinguish them from changes in coding practice, the timing of code registration, or postnatal case ascertainment. By contrast, the upward pattern in Turner and Klinefelter syndromes—particularly the post-2019 shifts—should be interpreted with caution. Although sex chromosome aneuploidies lack the early lethality or severe structural malformations characteristic of autosomal trisomies, their subtle phenotypes, such as developmental delays, stature variations, and atypical pubertal development, are being increasingly recognized. Nevertheless, these trends may partly reflect changes in administrative ascertainment under the RDCRP (specifically, the sequential inclusion of Down syndrome in 2004; Turner, Edwards, and Patau syndromes in 2009; and Klinefelter syndrome in 2019) rather than a true epidemiological increase in disease occurrence alone. Ultimately, further integrated analyses that account for shifting maternal age, administrative eligibility, and other demographic variables are warranted to precisely delineate the drivers of these diagnostic registration patterns.

This study has several limitations. First, as this study used administrative claims data, case identification relied on ICD-10 diagnostic codes and clinical registration rather than cytogenetic confirmation. Sex designations were based on the administrative records. The presence of male-coded patients among patients with Turner syndrome may reflect sex chromosome mosaicism, clerical errors, or registration issues. However, because this study used de-identified data, we could not verify karyotypes or perform individual-level adjudication [16,17,18]. Second, the claims-based diagnostic registration ratios reported in this study do not represent true cytogenetic birth prevalence. Since case ascertainment was based on the first appearance of disorder-specific ICD-10 codes in the HIRA claims database, the estimates should be interpreted as claims-based diagnostic registration ratios within the healthcare system. This distinction is particularly relevant for Turner and Klinefelter syndromes, which are often diagnosed during childhood, adolescence, and adulthood. Therefore, annual trends based on the year of diagnostic registration should be interpreted as changes in clinical recognition, healthcare utilization, and administrative registration rather than changes in biological disease occurrence alone. Third, data on prenatal diagnosis, pregnancy termination, and stillbirths were unavailable; thus, the observed claims-based registration ratios may have underestimated the true burden of chromosomal abnormalities, particularly in highly lethal trisomies. Fourth, mortality estimates were based on discharge-recorded in-hospital deaths captured in the claims data. Deaths occurring before diagnostic registration or outside hospital discharge records may have been missed, particularly among neonates with Edwards or Patau syndrome [6,7]. Fifth, because first-year healthcare expenditures and age at diagnosis showed skewed distributions, medians (Q1–Q3) were presented in addition to means. However, first-year healthcare expenditures were not age-adjusted and may partly reflect differences in diagnostic age rather than true disease-specific disparities. In addition, age in the HIRA database is recorded in years, and ages under 1 year are classified only as ≤28 days (0.2 years) or 29 days to <1 year (0.9 years); therefore, the reported mean and median ages at diagnosis should be regarded as approximate. Sixth, because disorders typically recognized later in childhood have shorter observation windows in recent birth cohorts (e.g., those born close to 2023), they are likely to be under-ascertained in these cohorts, and registration frequencies for recent years represent incomplete, lower-bound estimates. Finally, first-year healthcare expenditures showed substantial variability, and detailed expenditure categories could not be analyzed in this study. Therefore, comparisons based solely on mean total costs should be interpreted with caution, and the specific drivers of healthcare expenditure could not be evaluated. Future studies incorporating category-specific expenditure data are warranted to better characterize healthcare resource utilization and inform health policy and economic evaluations.

In conclusion, this study provides a comprehensive analysis of HIRA-based diagnostic registration patterns, temporal trends, and the multifaceted medical burdens of five selected common aneuploidies in South Korea. By defining diagnostic registration based on the first recorded clinical claim, this study offers a real-world perspective of how these conditions are captured within the Korean healthcare system. These findings highlight substantial disorder-specific differences in diagnostic timing, claims-based clinical and healthcare utilization burden, mortality, and healthcare expenditure. A national strategy that integrates early diagnosis, congenital anomaly surveillance, long-term care, and targeted financial support is essential for optimizing care pathways and resource allocation for affected children and families. Future research should address several remaining gaps. First, linking claims data with prenatal screening results, cytogenetic confirmation, pregnancy outcomes, and national mortality records would allow true birth prevalence and survival to be estimated more accurately than is possible with claims-based diagnostic registration and discharge-recorded mortality alone. Second, longitudinal follow-up into adulthood is needed, particularly for Turner and Klinefelter syndromes, whose diagnoses and major morbidities may emerge beyond childhood. Third, category-specific and broader societal cost analyses, including caregiver burden and productivity loss, would help identify the major drivers of economic burden and inform healthcare planning. Finally, the Korean experience with the RDCRP illustrates the potential value of linking financial support programs for rare diseases with nationwide diagnostic registration systems. Such frameworks may both facilitate access to care and support population-level surveillance, and may therefore provide a practical model for settings where dedicated congenital anomaly registries are not yet available.

## Figures and Tables

**Table 1:** Baseline Characteristics and First-Year Healthcare Expenditure of Patients with Selected Common Aneuploidies.

Variable	Down Syndrome	Edwards Syndrome	Patau Syndrome	Turner Syndrome	Klinefelter Syndrome
No. of patients	3511	273	91	1154	871
Sex (M/F), *n*	1923/1588	96/177	39/52	93/1061	867/4
Age at diagnosis, mean (SD), yr	0.69 (1.09)	0.75 (1.52)	1.71 (2.80)	4.57 (3.47)	3.03 (3.58)
Age at diagnosis, median (Q1–Q3), yr	0.2 (0.2–0.9)	0.2 (0.2–0.9)	0.2 (0.2–2)	4 (0.9–7)	0.9 (0.9–5)
Epilepsy, *n* (%) [95% CI]	116 (3.30) [2.74–3.95]	34 (12.45) [8.78–16.97]	15 (16.48) [9.53–25.73]	25 (2.17) [1.41–3.18]	14 (1.61) [0.88–2.68]
CHD, *n* (%) [95% CI]	2130 (60.67) [59.03–62.29]	188 (68.86) [63.01–74.31]	64 (70.33) [59.84–79.45]	132 (11.44) [9.66–13.42]	98 (11.25) [9.23–13.54]
First-year healthcare expenditure, mean (SD), KRW	12,455,946 (29,648,879)	52,998,814 (88,222,132)	37,897,745 (74,258,965)	2,853,011 (8,473,788)	1,503,182 (5,395,546)
First-year healthcare expenditure, median (Q1–Q3), KRW	2,898,565 (521,720–13,467,270)	17,394,435 (1,087,560–61,040,440)	7,478,665 (510,510–37,524,080)	555,270 (222,890–2,957,070)	242,990 (85,015–453,780)

CHD, congenital heart disease; CI, confidence interval; KRW, Korean won; SD, standard deviation; Q1–Q3, first to third quartile. Epilepsy and CHD are presented with exact (Clopper–Pearson) 95% CIs. First-year healthcare expenditure indicates the total healthcare expenditure within 365 days of the first observed claims-code date and was calculated for patients with a complete 365-day follow-up. The first-year healthcare expenditure analysis was restricted to patients whose first observed claims-code date occurred between 2009 and 2022 to ensure a complete 1-year follow-up; patients first coded in 2023 (*n* = 381) were excluded. The numbers of patients included in the expenditure analysis were as follows: Down syndrome, 3386; Edwards syndrome, 254; Patau syndrome, 86; Turner syndrome, 1025; and Klinefelter syndrome, 768 (total, 5519).

## Data Availability

The de-identified individual-level data used in this study are not publicly available due to data-use restrictions imposed by the Health Insurance Review and Assessment Service (HIRA) of Korea. Access to the data is subject to approval by HIRA in accordance with its data access policies.

## Supplementary Materials

The following supporting information can be downloaded at https://www.mdpi.com/article/10.3390/children13101360/s1. Table S1: Claims-based diagnostic registration frequency of selected common aneuploidies and the annual number of live births used as the denominator, Korea, 2009–2023; Table S2: Birth cohort-based diagnostic registration frequency of selected common aneuploidies, by year of birth, Korea, 2009–2023; Table S3: Sensitivity analysis of the sex chromosome aneuploidies excluding individuals whose recorded sex was discordant with the disorder.
