# Supplementary material for: Claims-Based Diagnostic Registration Patterns and Clinical Burden of Selected Common Aneuploidies in Korean Children: A Nationwide HIRA Study (2009–2023)
Source: Children (Basel). 2026 Oct 8;13(10):1360. doi: 10.3390/children13101360 (PMC13703676; doi:10.3390/children13101360)
Supplement: Supplementary file 1 [file children-13-01360-s001.zip › children-4526886-supplementary.pdf]

Supplementary Tables

Claims-Based Diagnostic Registration Patterns and Clinical Burden of Selected Common Aneuploidies in Korean Children: A Nationwide HIRA Study (2009–2023)

Table S1. Claims-based diagnostic registration frequency of selected common aneuploidies and the annual number of live births used as the denominator, Korea, 2009–2023.

Panel A. Disorder-specific registration frequency over the whole study period.

Disorder	Registered individuals (n)	Live births (N)	Per 100,000 live births	Registration ratio
Down syndrome	3511	5,586,770	62.8	1 per 1591
Edwards syndrome	273	5,586,770	4.9	1 per 20,464
Patau syndrome	91	5,586,770	1.6	1 per 61,393
Turner syndrome	1154	5,586,770	20.7	1 per 4841
Klinefelter syndrome	871	5,586,770	15.6	1 per 6414
**All five disorders**	**5900**	**5,586,770**	**105.6**	**1 per 947**

The registration frequency is an administrative registration ratio, not an incidence, prevalence, or birth prevalence. Numerator: individuals born in or after 2009 with at least one disorder-specific ICD-10 code (Q90.0–Q90.9, Q91.0–Q91.3, Q91.4–Q91.7, Q96.0–Q96.9, Q98.0–Q98.4) registered during 2009–2023. Denominator: all live births in Korea during 2009–2023.

Panel B. Annual number of live births used as the denominator.

Year	Live births	Year	Live births
2009	444,849	2017	357,771
2010	470,171	2018	326,822
2011	471,265	2019	302,676
2012	484,550	2020	272,337
2013	436,455	2021	260,562
2014	435,435	2022	249,186
2015	438,420	2023	230,028
2016	406,243	**Total**	**5,586,770**

Source: Statistics Korea, vital statistics of live births.

Table S2. Birth cohort-based diagnostic registration frequency of selected common aneuploidies, by year of birth, Korea, 2009–2023.

Birth year	Live births	Down	Edwards	Patau	Turner	Klinefelter
2009	444,849	469 (105.4)	20 (4.5)	11 (2.5)	109 (24.5)	59 (13.3)
2010	470,171	295 (62.7)	18 (3.8)	5 (1.1)	112 (23.8)	58 (12.3)
2011	471,265	325 (69.0)	25 (5.3)	7 (1.5)	138 (29.3)	68 (14.4)
2012	484,550	341 (70.4)	18 (3.7)	8 (1.7)	136 (28.1)	77 (15.9)
2013	436,455	313 (71.7)	20 (4.6)	9 (2.1)	119 (27.3)	62 (14.2)
2014	435,435	287 (65.9)	23 (5.3)	2 (0.5)	107 (24.6)	64 (14.7)
2015	438,420	274 (62.5)	19 (4.3)	8 (1.8)	99 (22.6)	61 (13.9)
2016	406,243	236 (58.1)	20 (4.9)	7 (1.7)	76 (18.7)	56 (13.8)
2017	357,771	215 (60.1)	22 (6.1)	10 (2.8)	58 (16.2)	66 (18.4)
2018	326,822	167 (51.1)	18 (5.5)	7 (2.1)	53 (16.2)	42 (12.9)
2019	302,676	149 (49.2)	25 (8.3)	5 (1.7)	36 (11.9)	63 (20.8)
2020	272,337	117 (43.0)	16 (5.9)	3 (1.1)	36 (13.2)	53 (19.5)
2021	260,562	130 (49.9)	10 (3.8)	1 (0.4)	34 (13.0)	48 (18.4)
2022	249,186	92 (36.9)	7 (2.8)	7 (2.8)	23 (9.2)	44 (17.7)
2023	230,028	101 (43.9)	12 (5.2)	1 (0.4)	18 (7.8)	50 (21.7)
**Total**	**5,586,770**	**3511 (62.8)**	**273 (4.9)**	**91 (1.6)**	**1154 (20.7)**	**871 (15.6)**

Values are n (per 100,000 live births of the same birth year). Year of birth was derived as (year of first observed claims-code date, and age recorded at that date); individuals born before 2009 were excluded because of left truncation. For each birth cohort the numerator is disorder-specific whereas the denominator—the number of live births in that birth year—is shared across disorders. The numerator set is identical to that of the main analysis and only its distribution across years differs, so the overall frequency is unchanged from Supplementary Table S1.

Observation time differs systematically across birth cohorts: children born in 2009 could be followed for up to 15 years, whereas those born in 2023 could be followed for at most 1 year. Estimates for the most recent birth cohorts are therefore incomplete and should be regarded as lower bounds, particularly for Turner and Klinefelter syndromes, in which diagnosis frequently occurs several years after birth.

Table S3. Sensitivity analysis of the sex chromosome aneuploidies excluding individuals whose recorded sex was discordant with the disorder.

	**Turner, all**	**Turner, female only**	**Klinefelter, all**	**Klinefelter, male only**
Individuals, n	1154	1061	871	867
Excluded records, n (%)	–	93 (8.1)	–	4 (0.5)
Registration frequency, per 100,000 live births	20.7	19.0	15.6	15.5
Age at diagnosis, mean (SD), yr	4.57 (3.47)	4.81 (3.47)	3.03 (3.58)	3.04 (3.60)
Epilepsy, n (%)	25 (2.17)	21 (1.98)	14 (1.61)	14 (1.61)
Congenital heart disease, n (%)	132 (11.44)	123 (11.59)	98 (11.25)	98 (11.29)
Discharge-recorded in-hospital death, n (%)	1 (0.09)	1 (0.09)	0 (0.00)	0 (0.00)
First-year healthcare expenditure, mean (SD), KRW	2,853,011 (8,473,788)	2,840,103 (8,710,931)	1,503,182 (5,395,546)	1,506,791 (5,408,994)
First-year healthcare expenditure, median (Q1–Q3), KRW	555,270 (222,890–2,957,070)	568,150 (223,690–2,843,480)	242,990 (85,015–453,780)	242,990 (85,015–443,500)

Ninety-three individuals with a Turner syndrome code whose recorded sex was male (8.1% of 1154) and four individuals with a Klinefelter syndrome code whose recorded sex was female (0.5% of 871) were excluded. Individual-level karyotype results are not available in the HIRA claims database, so it cannot be determined whether these records represent mosaicism, coding or clerical errors, or administrative sex-registration issues. All principal estimates were essentially unchanged after exclusion.
